# Prediction of postpartum hemorrhage (PPH) using machine learning algorithms in a Kenyan population

**DOI:** 10.3389/fgwh.2023.1161157

**Published:** 2023-07-28

**Authors:** Santosh Yogendra Shah, Sumant Saxena, Satya Pavitra Rani, Naresh Nelaturi, Sheena Gill, Beth Tippett Barr, Joyce Were, Sammy Khagayi, Gregory Ouma, Victor Akelo, Errol R. Norwitz, Rama Ramakrishnan, Dickens Onyango, Manoj Teltumbade

**Affiliations:** ^1^ CognitiveCare Inc., Milpitas, CA, United States; ^2^ Office of the Director, Nyanja Health Research Institute, Salima, Malawi; ^3^ Center for Global Health Research, Kenya Medical Research Institute, Kisumu, Kenya; ^4^ Center for Global Health, U.S. Centers for Disease Control and Prevention, Kisumu, Kenya; ^5^ Department of Obstetrics and Gynecology, Tufts University School of Medicine, Boston, MA, United States; ^6^ Operations Research and Statistics, MIT Sloan School of Management, Cambridge, MA, United States; ^7^ Kisumu County Department of Health, Kisumu, Kenya

**Keywords:** maternal health, machine learning, pregnancy, postpartum hemorrhage, risk prediction, LMICs

## Abstract

**Introduction:**

Postpartum hemorrhage (PPH) is a significant cause of maternal mortality worldwide, particularly in low- and middle-income countries. It is essential to develop effective prediction models to identify women at risk of PPH and implement appropriate interventions to reduce maternal morbidity and mortality. This study aims to predict the occurrence of postpartum hemorrhage using machine learning models based on antenatal, intrapartum, and postnatal visit data obtained from the Kenya Antenatal and Postnatal Care Research Collective cohort.

**Method:**

Four machine learning models – logistic regression, naïve Bayes, decision tree, and random forest – were constructed using 67% training data (1,056/1,576). The training data was further split into 67% for model building and 33% cross validation. Once the models are built, the remaining 33% (520/1,576) independent test data was used for external validation to confirm the models' performance. Models were fine-tuned using feature selection through extra tree classifier technique. Model performance was assessed using accuracy, sensitivity, and area under the curve (AUC) of the receiver operating characteristics (ROC) curve.

**Result:**

The naïve Bayes model performed best with 0.95 accuracy, 0.97 specificity, and 0.76 AUC. Seven factors (anemia, limited prenatal care, hemoglobin concentrations, signs of pallor at intrapartum, intrapartum systolic blood pressure, intrapartum diastolic blood pressure, and intrapartum respiratory rate) were associated with PPH prediction in Kenyan population.

**Discussion:**

This study demonstrates the potential of machine learning models in predicting PPH in the Kenyan population. Future studies with larger datasets and more PPH cases should be conducted to improve prediction performance of machine learning model. Such prediction algorithms would immensely help to construct a personalized obstetric path for each pregnant patient, improve resource allocation, and reduce maternal mortality and morbidity.

## Introduction

Postpartum hemorrhage (PPH) is a major cause of maternal mortality worldwide, accounting for 30%–50% of maternal deaths (1–3). Most maternal deaths (99%) occur in low- and middle-income countries (LMICs) (2). Sub-Saharan Africa is disproportionately affected, with the highest maternal mortality (66% of global burden) (4) and a PPH prevalence of 10.5% (5), accounting for 1 in 4 maternal deaths (1) PPH is also linked to severe maternal morbidity, including puerperal hysterectomy, multiple organ failure, and chronic psychological trauma (6, 7).

Despite aggressive governmental efforts over the last 15 years to reduce maternal mortality in Kenya, including the implementation of a reproductive health voucher program in 2006 (8) and the provision of free maternity treatments in government facilities in 2013 (9), progress has been slow. The maternal mortality ratio (MMR) in Kenya is high (342 per 100,000 live births) compared to the current global MMR of 211 per 100,00 live births (4). A reduction in maternal mortality to a target of less than 70 maternal deaths per 100,000 live births is one of the United Nations' Sustainable Development Goals for 2030 (10). The ability to identify patients at risk of PPH and associated complications reliably, accurately, and early in pregnancy would be an important step towards achieving this aspirational goal.

In contrast to traditional general-purpose predictive algorithms, which merely transform input data into an output based on predetermined rules, artificial intelligence (AI) systems can generate new rules and patterns by analyzing both input and output data. A recent systematic review found three PPH risk prediction models with promising clinical applications (11), but AI approaches have not yet been thoroughly evaluated in obstetrics (12). Moreover, these predictive models that have been developed thus far used populations from the United Kingdom (13), South Korea (14), and China (15), and focused on PPH in the setting of cesarean delivery. Predictive modeling for PPH in a general obstetric LMICs population has not previously been reported.

The aim of this study was to construct and validate machine learning models to predict PPH in a general obstetric population using data from the Kenya Antenatal and Postnatal Care Research Collective (ARC) cohort. The long-term goal is to allow obstetricians to identify patients at high risk of PPH and guide clinical decision-making.

## Materials and methods

### Study population

Data from the Maternal and Newborn Health (MNH) monitoring report collected by ARC were utilized for the development and validation of PPH prediction models. Briefly, MNH monitoring report data were collected as a part of prospective longitudinal study for pregnancy risk stratification innovation and measurement alliance at multiple LMIC sites, including in Ghana, Kenya, Zambia, and Pakistan. For this study, we used the antenatal, intrapartum, and postnatal visit data from the Kenya site collected between August 2020 and February 2022. The inclusion criteria were women with documented antenatal, intrapartum, and postnatal visits, including delivery outcome and reported PPH outcome. As a part of Maternal labor and delivery outcome documentation, PPH outcome were reported by healthcare staff within 24 h of delivery. Data were collected during home visits by healthcare staff as well as during healthcare facility visits, including antenatal clinic (ANC) visits, intrapartum visit within 24 h of delivery, and postnatal care (PNC) visits.

### Experimental design

Demographic and clinical information collected during the ANC and intrapartum (delivery) visits were used to build the predictive models. The data were randomly divided into a training (67%) and independent testing (33%) dataset. The ratio of PPH to non-PPH cases was kept similar in training and testing dataset. The training dataset was again randomly divided into 67% and 33%, and the smaller dataset used for cross validation. The definition and classification of PPH in the literature is variable, depending on such factors as the estimated blood loss (EBL) (>500 ml or >1,000 ml), type of delivery (vaginal vs. cesarean), and timing of hemorrhage (early vs. late) (16–18). In this study, we used the outcome of interest (presence or absence of PPH) as reported by healthcare staff while collecting maternal labor and delivery outcome during delivery visit. This was generally determined using the criteria of EBL > 500 ml after vaginal delivery and >1,000 ml after cesarean delivery. All were early PPH cases as they were documented at or shortly after delivery.

### Feature engineering and machine learning

Data were collected across four ANC visits at 0–17 weeks, 18–25 weeks, 26–33 weeks, and ≥34 weeks of gestational age. Socio-demographic data (such as age and height) were collected during the first ANC visit, whereas clinical data (such as hemoglobin level, blood pressure, and proteinuria,) were collected at each ANC visit. Overall, around 700 features consisting of categorical, numerical, and date/time variables were collected from each study subject across all pregnancy visits. To develop predictive algorithms for PPH, most of the features collected after the onset of labor (with the exception of the presence or absence of PPH) and all of the features collected at the PNC visit were removed from the analysis. The exclusion of such features was intentional to facilitate early prediction of PPH. This approach aims to optimize resource allocation, particularly in low-resource healthcare settings prevalent in LMICs. When two columns or features were highly correlated with each other as identified by data science techniques, only one of them was retained to avoid multicollinearity. Missing values were handled in one of two ways: for categorical variables, a new category “others” was created; for numerical variables, missing values were imputed using Generative Adversarial Nets Framework (GAIN) imputation methodology (19).

Data for each study subject were captured at different gestational ages: 47% of women had their first ANC visit at 0–13 weeks of gestation, whereas 53% had their first ANC visit at 14–18 weeks of gestation. This is common in the healthcare setting resulting in data that is heterogenous and irregularly sampled at multiple time points. To address this issue, we employed the FIDDLE (Flexible Data-Driven Pipeline) framework (20) and transformed our features into two categories: time-invariant and time-variant features. Time-invariant features are those that were collected only once and typically do not vary (such as maternal age), whereas time-variant features were collected at different time points and vary over the course of the study (such as blood pressure).

### Model performance and comparison

First, we trained four machine learning models (logistic regression, naïve Bayes, decision tree, and random forest) to predict PPH outcome using all of the features. The internal validation was performed using k-fold cross-validation. Thereafter, secondary models were built using limited sets of features selected through extra trees classifier, thereby making it more relevant to the clinical setting. For comparison between the models and to evaluate performance accuracy, the sensitivity, and area under the curve (AUC) of the receiver operating characteristics (ROC) curve were calculated using model predictions on the independent testing dataset.

### Model interpretation

To estimate relative relevance of each feature, Shapley values were calculated using python library SHAP (21). To understand the importance of each feature towards predicting PPH, the mean absolute SHAP (SHapley Additive exPlanations) values were plotted for each individual in the training dataset.

## Results

**Figure 1:**
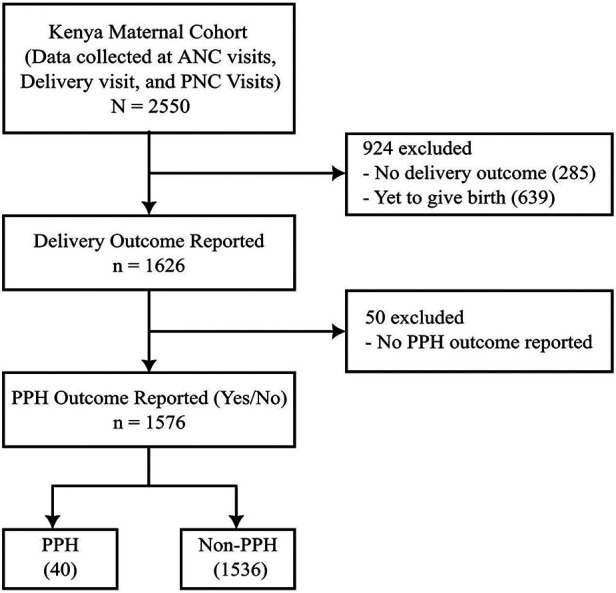
Flow chart of Kenya maternal cohort.

A total of 2,550 women were included in the Kenya maternal cohort. Of those, women were excluded if there was no reported delivery outcome (*n* = 924, 36.2%) or no report of the presence or absence of PPH (*n* = 50, 2.0%), leaving 1,576 women (61.8%) in the final analysis (Figure 1). Among these 1,576 women, 40 (2.5%) were reported to have PPH. Table 1 presents the comparison of demographic and clinical characteristics between the PPH and non-PPH groups, revealing no significant differences between two groups. The mean age was 28.5 and 26.4 years for the PPH and non-PPH groups, respectively.

**Table 1:** Demographic and clinical characteristics of the study populations.

	PPH (*n* = 40)	No PPH (*n* = 1,536)
Demographic Characteristics		
Maternal age, years	28.5 ± 6.8	26.4 ± 5.3
Vaginal delivery	33 (82.5%)	1,324 (86.2%)
Cesarean delivery	7 (17.5%)	212 (13.8%)
Gestational age at delivery, weeks	35.3 ± 7.2	36.6 ± 9
Previous pregnancies		
Nulligravida	14 (35%)	473 (30.8%)
≥1	26 (65%)	1,059 (68.9%)
Previous pregnancies with live birth		
0	1 (2.5%)	60 (4%)
1	10 (25%)	414 (27%)
≥2	15 (37.5%)	585 (38%)
Clinical Characteristics (Intrapartum)		
Systolic blood pressure, mmHg	119.9 ± 16.3	119.4 ± 15.6
Diastolic blood pressure, mmHg	75.3 ± 11.7	76.2 ± 11.3
Temperature, °C	36.4 ± 0.4	36.4 ± 0.4
Total hemoglobin, g/dl	12.4 ± .6	12.3 ± 1.4
Heart rate, beats/minute	88.4 ± 13.3	86.9 ± 13.3
Respiratory rate, breaths per minute	19.0 ± 2.6	21.2 ± 3.7
Oxygen saturation, %	97.3 ± 1.4	97.8 ± 1.3

Data are given as *n* (%) or mean ± SD. (PPH, postpartum hemorrhage).

**Figure 2:**
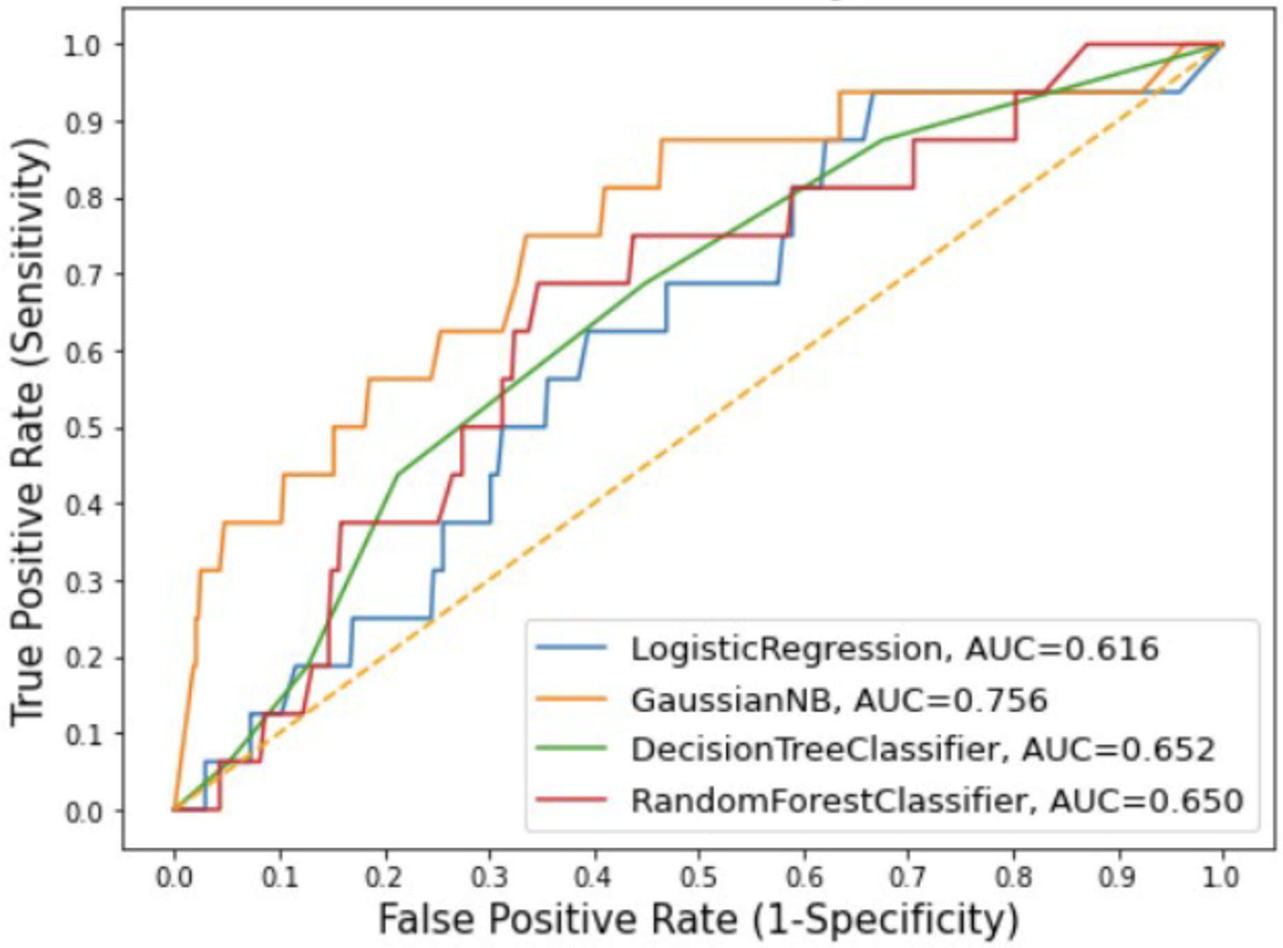
Receiver operating characteristics curves of four machine learning model of PPH prediction (AUC, area under the curve; GaussianNB, naïve Bayes).

A total of 58 candidate features were derived from the overall 707 features and used to develop the PPH prediction models. The features included, amongst others, maternal age, gestational age, hemoglobin levels, systolic and diastolic blood pressure, and respiratory rate. These 58 features were transformed into 264 variables based on the time at which the variables were captured. Using these variables, logistic regression, naïve Bayes, decision tree, and random forest models were built. Due to high-class imbalance (40 PPH cases and 1,536 non-PPH cases), the logistic model did not perform well with AUC of 0.51 on the testing dataset. The remaining three models performed marginally better, with random forest and naïve Bayes both having AUC of 0.55 on the testing dataset (Table 2). To further improve the performance of the models, the top 10 variables were selected using the extra trees classifier technique. Out of these top 10 variables, few were associated with the following features collected at intrapartum period: systolic blood pressure, diastolic blood pressure, respiratory rate, hemoglobin levels, and signs of pallor. Remaining variables were related with features such as time of third ANC visit, anemia diagnosis at first trimester and second trimester, and fetal heart rate in third trimester. As compared to the baseline models, the performance of all the models improved after training on these 10 selected variables. The naïve Bayes model performed significantly better than the other models when compared across the majority of performance metrics with 0.76 AUC, 0.95 accuracy, and 0.97 specificity (Table 3). The comparison of ROC curves across the four models after training is shown in Figure 2.

**Table 2:** Performance matrix of machine learning models on independent test set before feature selection. (AUC, area under the curve).

Model	AUC	Accuracy	Sensitivity	Specificity
Logistic Regression	0.51	0.72	0.31	0.74
Naïve Bayes	0.55	0.64	0.50	0.64
Decision Tree	0.53	0.50	0.56	0.50
Random Forest	0.55	0.89	0.13	0.90

**Table 3:** Performance matrix of machine learning models on independent test set after feature selection using extra tree classifier technique. (AUC, area under the curve).

Model	AUC	Accuracy	Sensitivity	Specificity
Logistic Regression	0.62	0.65	0.50	0.66
Naïve Bayes	0.76	0.95	0.31	0.97
Decision Tree	0.65	0.62	0.69	0.55
Random Forest	0.65	0.82	0.37	0.78

**Figure 3:**
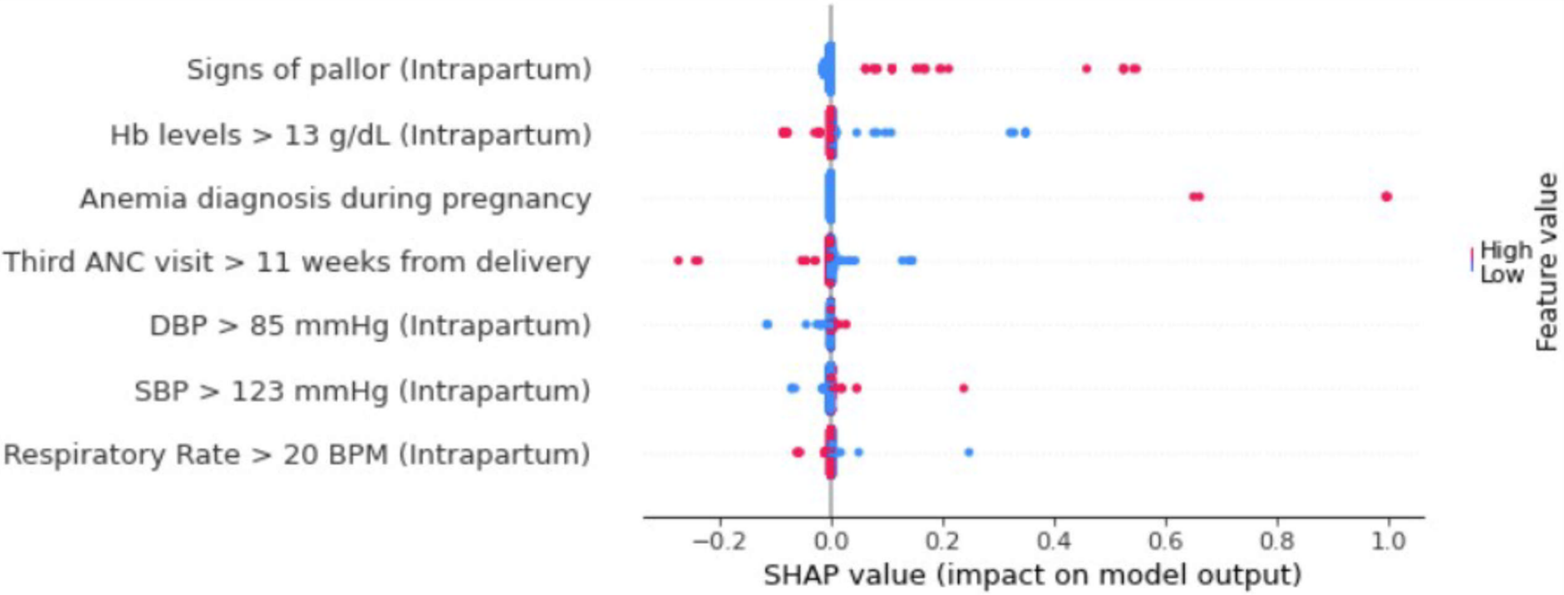
SHAP summary plot of top 7 features for naïve Bayes PPH prediction model. Color represents relative feature value for each patient. Blue color indicates low feature value whereas red color indicates high feature value. Positive SHAP values suggest greater PPH risk and negative SHAP values are protective for PPH (Hb, hemoglobin; ANC, antenatal clinic; SBP, systolic blood pressure; DBP, diastolic blood pressure; BPM, breaths per minute, SHAP, Shapley Additive exPlanations).

As the naïve Bayes model performed best, we performed SHAP analysis using this model to investigate the impact of individual variables on PPH prediction. The critical features associated with a high risk of PPH included: (1) signs of pallor documented during the intrapartum visit, (2) a diagnosis of anemia made anytime during the pregnancy (defined as hemoglobin levels less than 11 g/dl), (3) limited prenatal care (defined as the third ANC visit occurring within 11 weeks of delivery), (4) elevated diastolic blood pressure at intrapartum visit (greater than 85 mmHg), and (5) elevated systolic blood pressure at intrapartum visit (greater than 123 mmHg). In contrast, elevated hemoglobin concentrations at intrapartum visit (greater than 13 g/dl) and rapid respiratory rate (more than 20 breaths per minute at intrapartum visit) were protective of PPH prediction (Figure 3).

## Discussion

### Main findings

In this study, we demonstrated that machine learning can be employed to predict PPH using routine clinical data collected at routine antenatal and intrapartum visits. The ability to accurately predict patients at high risk of PPH is important in order to identify and stratify their care and mitigate the consequences of this dangerous condition. The association of clinical features such as blood pressure, respiratory rate, hemoglobin levels, and anemia with the development of PPH is consistent with published data (6, 22). The association with anemia is particularly significant since, in addition to predisposing women to PPH, it also limits tolerance to blood loss (18). Our final model did not include many previously identified risk factors such as a history of PPH in a prior pregnancy, route of delivery, and multiple gestations. This could be because of the low incidence of such conditions in the study population or because the drivers and risk factors for PPH may be different in a LMIC population such as that in Kenya as compared to a western population.

In this cohort, we also found an association between ANC visits and risk of PPH. More limited prenatal care (defined as the third ANC visit occurring within 11 weeks of delivery) was associated with an increased risk of PPH. This underlines the importance of prenatal care and timely ANC visits in LMICs. Such insights can help inform policies that address unfavorable social determinants of health to take down barriers that prevent some women from accessing care and adhering to recommended antenatal visit schedules. This finding further suggests that targeted interventions to improve access and visit compliance may reduce maternal mortality and morbidity due to PPH in LMICs.

In our study, we made notable observations regarding the performance of different classifiers on an independent test dataset. Specifically, the naïve Bayes classifier exhibited superior accuracy compared to random forest and decision tree, which experienced significant decline in accuracy. This decline may indicate a potential issue of overfitting in the latter classifiers (23, 24). In contrast, recent study on Iran population found that machine learning model such as random forest and decision tree provided improved performance in predicting PPH (25). Westcott et al. (26) also reported better performance of boosted decision trees for prediction of PPH in United States population. Additionally, we observed suboptimal performance of logistic regression when compared to naïve Bayes. This can be attributed to the relatively small sample size of the PPH population. Previous studies have consistently demonstrated that generative classifiers such as naïve Bayes tend to outperform discriminatory classifiers like logistic regression when trained on limited sample sizes (27, 28).

### Clinical implications

These findings suggest that routine clinical and demographics data can be used to predict women at high risk for adverse pregnancy events. At present, healthcare providers predominantly rely on clinical expertise and nonspecific risk factors to identify high-risk pregnancies. Further, the International Federation of Gynecology and Obstetrics (FIGO) developed a PPH care pathway to integrate WHO PPH guidelines in Sub-Saharan African countries (29). These guidelines are primarily derived from expert opinion and clinical consensus lacking the ability to offer personalized risk predictions. By incorporating such predictive models into clinical practice, healthcare professionals will be able to personalize and stratify the care of women at high-risk for PPH. For such cases, targeted interventions may include treating anemia, administrating prophylactic medication (often referred to as active management of the third stage of labor), ensuring the availability of blood products during delivery, and/or non-clinical interventions such as providing free transportation to the clinic or home visits in resource-limited settings. The efficacy of these intervention modalities should be tested against existing practice before introducing them into routine clinical practice. This can be accomplished with impact studies such as cluster randomized trials (30, 31).

### Strengths and limitations

To our knowledge, this is the first study to predict PPH in a population from Sub-Saharan Africa using a machine learning approach. This “proof of concept” study demonstrates that—even in LMICs with limited resources and a lack of standardized electronic health records—such predictive machine learning techniques can be used to identify women at high-risk of serious adverse pregnancy events, such as PPH which is a major cause of maternal mortality and morbidity. Additional studies are needed to prospectively validate our model and to determine whether it is generalizable to other Sub-Saharan African populations.

Limitations of this study include a small sample size with only 40 PPH cases. Moreover, our cohort had a PPH rate of 2.5%, which is significantly lower than the reported 10.5% prevalence of PPH in this region (5). The exclusion of late PPH cases, considering that the PPH outcome was reported within 24-hours of delivery, could potentially account for the lower PPH rate observed in the present study population. Additionally, it is plausible that the underreporting of cases could be another contributing factor to the observed low PPH rate. Data quality with a high proportion of missing data is another limitation of this study. Given the current state of clinical documentation in LMICs, it is possible that certain important factors were not captured and therefore could not be incorporated into the model. Future studies including larger datasets with more cases of PPH would allow for the careful examination and inclusion of additional variables into the prediction models for improved prediction performance.

## Conclusions

Seven factors (anemia, limited prenatal care, hemoglobin concentrations, signs of pallor at intrapartum, intrapartum systolic blood pressure, intrapartum diastolic blood pressure, and intrapartum respiratory rate) were associated with PPH prediction in Kenyan population. These findings provide an opportunity to explore machine learning approaches to identify patients at high-risk of PPH in resource constrained settings. Use of such predictive models to identify and stratify women at high risk of PPH and other adverse pregnancy events could bring us one step closer to designing a personalized obstetric journey for each pregnant patient, improving resource allocation, and ultimately reducing maternal mortality and morbidity.

## Data Availability

As the raw data supporting the conclusions of this article contains patient-level information, it is available upon request to the authors.

## References

[1] Say L Chou D Gemmill A Tunçalp Ö Moller AB Daniels J Global causes of maternal death: a WHO systematic analysis. Lancet Glob Health. (2014) 2(6):e323–33. 10.1016/S2214-109X(14)70227-X 25103301

[2] Ronsmans C Graham WJ. Maternal mortality: who, when, where, and why. Lancet. (2006) 368(9542):1189–200. 10.1016/S0140-6736(06)69380-X 17011946

[3] Khan KS Wojdyla D Say L Gülmezoglu AM van Look PF. WHO Analysis of causes of maternal death: a systematic review. Lancet. (2006) 367(9516):1066–74. 10.1016/S0140-6736(06)68397-9 16581405

[4] World Health Organization. Trends in maternal mortality 2000–2017: estimates by WHO, UNICEF, UNFPA, World Bank Group and the United Nations Population Division (2019). Available at: https://apps.who.int/iris/handle/10665/327595 (Accessed November 21, 2022).

[5] Carroli G Cuesta C Abalos E Gulmezoglu AM. Epidemiology of postpartum haemorrhage: a systematic review. Best Pract Res Clin Obstet Gynaecol. (2008) 22(6):999–1012. 10.1016/j.bpobgyn.2008.08.004 18819848

[6] Sebghati M Chandraharan E. An update on the risk factors for and management of obstetric haemorrhage. Womens Health (Lond). (2017) 13(2):34–40. 10.1177/1745505717716860 28681676 PMC5557181

[7] Sentilhes L Gromez A Clavier E Resch B Descamps P Marpeau L. Long-term psychological impact of severe postpartum hemorrhage. Acta Obstet Gynecol Scand. (2011) 90(6):615–20. 10.1111/j.1600-0412.2011.01119.x 21370999

[8] Amendah DD Mutua MK Kyobutungi C Buliva E Bellows B. Reproductive health voucher program and facility based delivery in informal settlements in Nairobi: a longitudinal analysis. PLoS One. (2013) 8(11):e80582. 10.1371/journal.pone.0080582 24260426 PMC3832453

[9] Gitobu CM Gichangi PB Mwanda WO. The effect of Kenya’s free maternal health care policy on the utilization of health facility delivery services and maternal and neonatal mortality in public health facilities. BMC Pregnancy Childbirth. (2018) 18(1):1–11.. 10.1186/s12884-018-1708-2 29580207 PMC5870237

[10] United Nations. Goal 3. Ensure healthy lives and promote well-being for all at all ages—Sustainable Development Goal Indicators. Available at: https://unstats.un.org/sdgs/report/2016/goal-03/. (Accessed November 21, 2022)

[11] Neary C Naheed S McLernon DJ Black M. Predicting risk of postpartum haemorrhage: a systematic review. BJOG. (2021) 128(1):46–53. 10.1111/1471-0528.16379 32575159

[12] Escobar GJ Gupta NR Walsh EM Soltesz L Terry SM Kipnis P. Automated early detection of obstetric complications: theoretic and methodologic considerations. Am J Obstet Gynecol. (2019) 220(4):297–307. 10.1016/j.ajog.2019.01.208 30682365

[13] Dunkerton SE Jeve YB Walkinshaw N Breslin E Singhal T. Predicting postpartum hemorrhage (PPH) during cesarean delivery using the Leicester PPH predict tool: a retrospective cohort study. Am J Perinatol. (2018) 35(2):163–9. 10.1055/s-0037-1606332 28847038

[14] Kim JW Lee YK Chin JH Kim So Lee MY Won HS Development of a scoring system to predict massive postpartum transfusion in placenta previa totalis. J Anesth. (2017) 31(4):593–600. 10.1007/s00540-017-2365-8 28466102

[15] Wu Q Yao K Liu Z Li L Zhao X Wang S Radiomics analysis of placenta on T2WI facilitates prediction of postpartum haemorrhage: a multicentre study. EBioMedicine. (2019) 50:355–65. 10.1016/j.ebiom.2019.11.010 31767539 PMC6921361

[16] Wormer KC Jamil RT Bryant SB. Acute postpartum hemorrhage. In: *StatPearls*. Treasure Island (FL): StatPearls Publishing (2022). Available at: https://www.ncbi.nlm.nih.gov/books/NBK499988/. (Accessed December 2, 2022) 29763164

[17] ACOG Committee on Practice Bulletins-Obstetrics. Practice bulletin No. 183: postpartum hemorrhage. Obstet Gynecol. (2017) 130(4):e168–86. 10.1097/AOG.0000000000002351 28937571

[18] Mavrides E Allard S Chandraharan E Collins P Green L Hunt BJ Prevention and management of postpartum haemorrhage. BJOG. (2016) 124(5):e106–49. 27981719 10.1111/1471-0528.14178

[19] Yoon J Jordon J van der Schaar M. GAIN: missing data imputation using generative adversarial nets. International conference on machine learning. PMLR (2018). p. 5689–98. Available at: https://proceedings.mlr.press/v80/yoon18a.html (Accessed December 2, 2022)

[20] Tang S Davarmanesh P Song Y Koutra D Sjoding MW Wiens J. Democratizing EHR analyses with FIDDLE: a flexible data-driven preprocessing pipeline for structured clinical data. J Am Med Inform Assoc. (2020) 27(12):1921–34. 10.1093/jamia/ocaa139 33040151 PMC7727385

[21] Lundberg SM Lee SI. A unified approach to interpreting model predictions. In: Guyon I, Von Luxburg U, Bengio S, Wallach H, Fergus R, Vishwanathan S, et al. editors. *Advances in Neural Information Processing Systems 30 (NIPS 2017)*. (2017) 30, Available at: https://proceedings.neurips.cc/paper/2017/hash/8a20a8621978632d76c43dfd28b67767-Abstract.html. (Accessed June 27, 2023)28753540

[22] Zheutlin AB Vieira L Shewcraft RA Li S Wang Z Schadt E Improving postpartum hemorrhage risk prediction using longitudinal electronic medical records. J Am Med Inform Assoc. (2022) 29(2):296–305. 10.1093/jamia/ocab161 34405866 PMC8757294

[23] Tu JV. Advantages and disadvantages of using artificial neural networks versus logistic regression for predicting medical outcomes. J Clin Epidemiol. (1996) 49(11):1225–31. 10.1016/S0895-4356(96)00002-9 8892489

[24] Hastie T Tibshirani R Friedman JH Friedman JH. The elements of statistical learning: Data mining, inference, and prediction. New York: Springer (2009).

[25] Mehrnoush V Ranjbar A Farashah MV Darsareh F Shekari M Jahromi MS. Prediction of postpartum hemorrhage using traditional statistical analysis and a machine learning approach. AJOG Global Reports. (2023) 3(2):100185. 10.1016/j.xagr.2023.100185 36935935 PMC10020099

[26] Westcott JM Hughes F Liu W Grivainis M Hoskins I Fenyo D. Prediction of maternal hemorrhage using machine learning: retrospective cohort study. J Med Internet Res. (2022) 24(7):e34108. 10.2196/34108 35849436 PMC9345059

[27] Ng A Jordan M. On discriminative vs. Generative classifiers: a comparison of logistic regression and naive Bayes. In: Dietterich T, Becker S, Ghahramani Z, editors. *Advances in Neural Information Processing Systems*. (Vol. 14). MIT Press. (2001) Available at: https://papers.nips.cc/paper/2001/file/7b7a53e239400a13bd6be6c91c4f6c4e-Paper.pdf (Accessed June 27, 2023).

[28] Huang Y Li L. Naive Bayes classification algorithm based on small sample set. 2011 IEEE international conference on cloud computing and intelligence systems. IEEE (2011). p. 34–9

[29] Ameh CA Meka RJ West F Dickinson F Allott H Godia P. A synthesis of clinical and health system bottlenecks to implementing new WHO postpartum hemorrhage recommendations: secondary data analysis of the Kenya confidential enquiry into maternal deaths 2014–2017. Int J Gynaecol Obstet. (2022) 158:14–22. 10.1002/ijgo.14270 35762810 PMC9544179

[30] Grant SW Collins GS Nashef SAM. Statistical primer: developing and validating a risk prediction model. Eur J Cardiothorac Surg. (2018) 54(2):203–8. 10.1093/ejcts/ezy180 29741602

[31] Moons KG Altman DG Vergouwe Y Royston P. Prognosis and prognostic research: application and impact of prognostic models in clinical practice. Br Med J. (2009) 338:606. 10.1136/bmj.b606 19502216

